# Supplementary figures and images for: Exceptionally preserved ‘skin’ in an Early Cretaceous fish from Colombia
Source: PeerJ. 2020 Jul 8;8:e9479. doi: 10.7717/peerj.9479 (PMC7353916; doi:10.7717/peerj.9479)

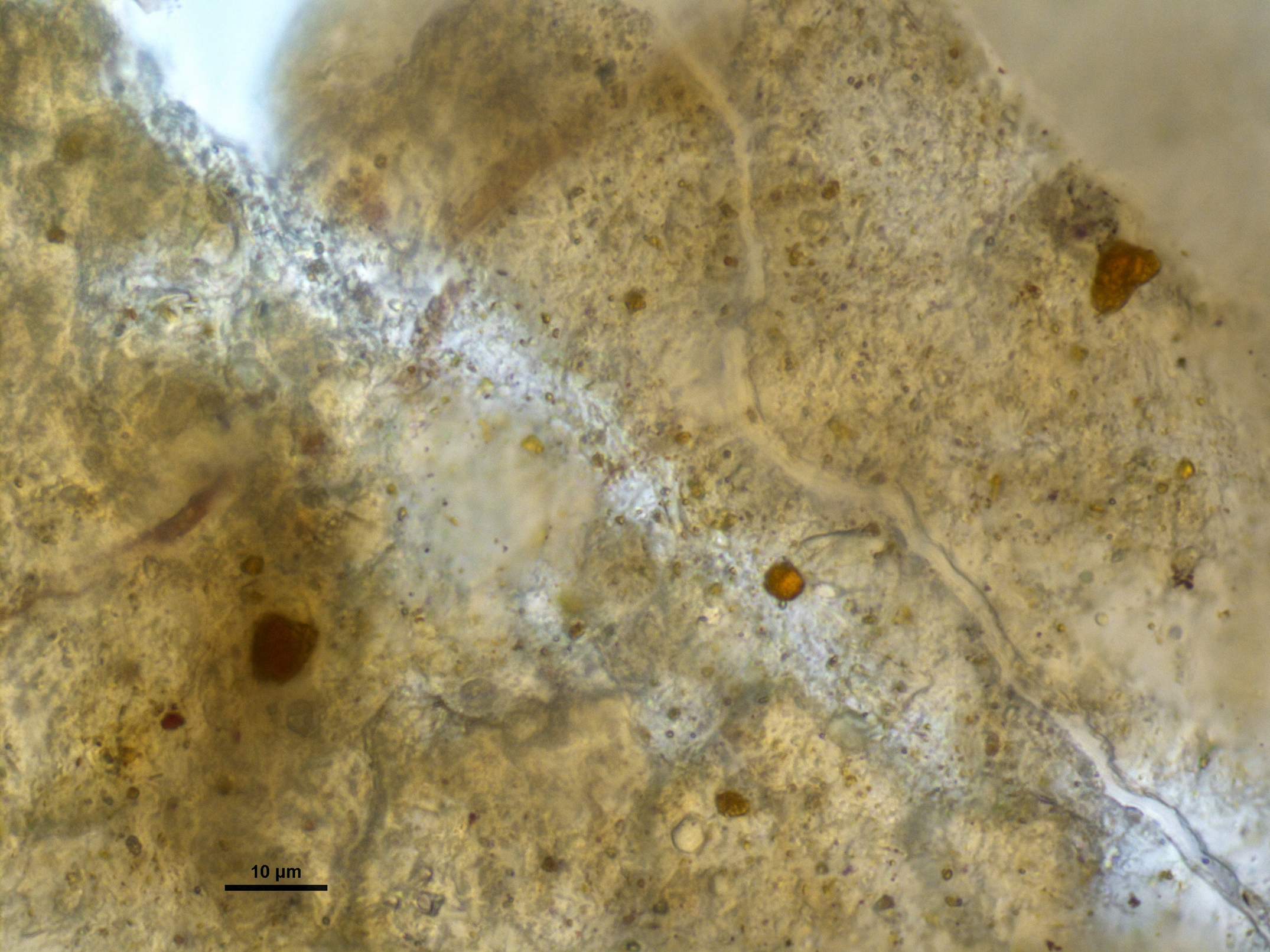

Supplement: Supplemental Information 3 [file peerj-08-9479-s003.png]

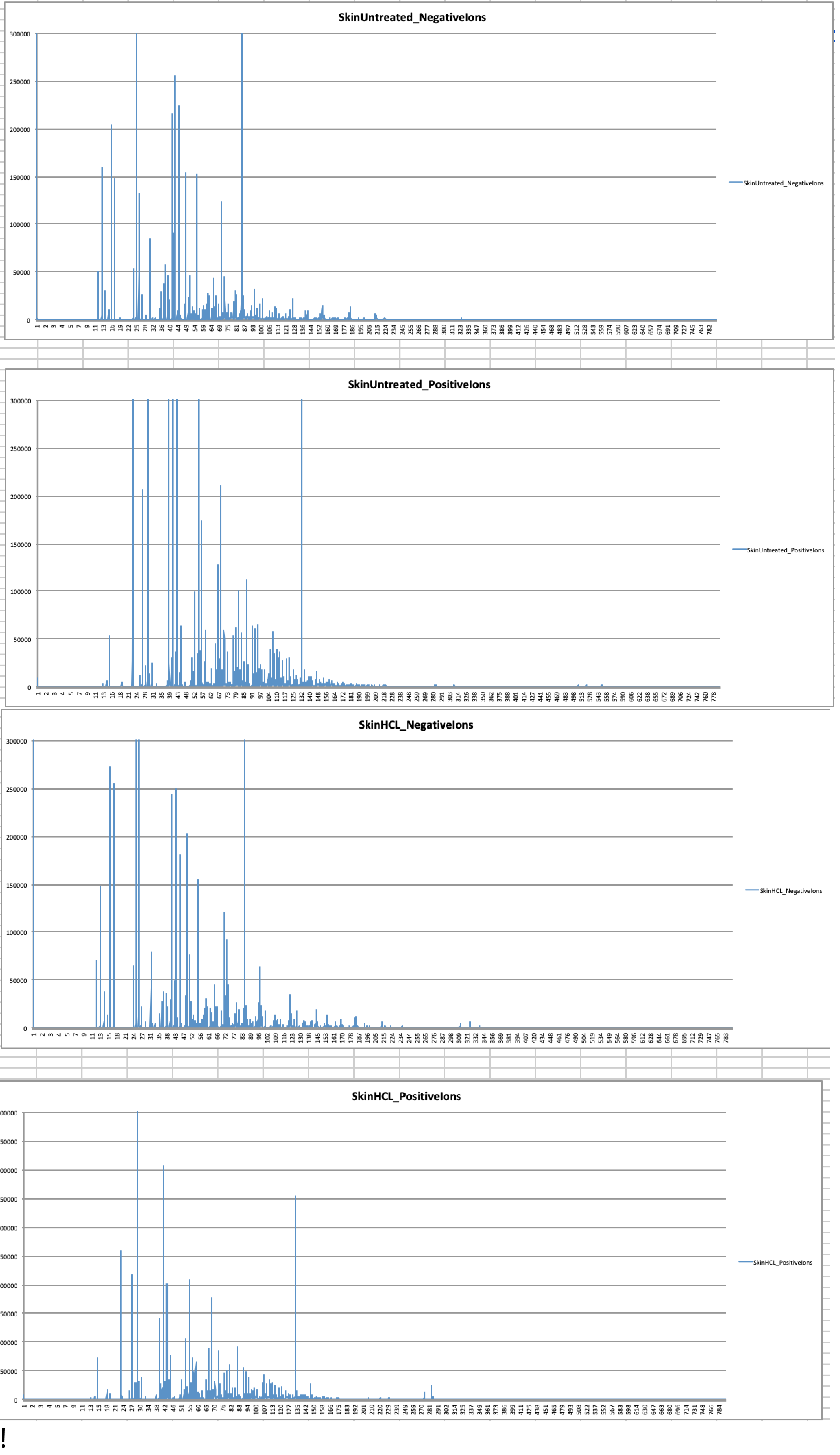

Supplement: Supplemental Information 4 — Top. Negative and positive ions spectra for untreated skin sample. Bottom. Negate and positive ions spectra for HCl treated skin sample. Also see Data S1. [file peerj-08-9479-s004.png]

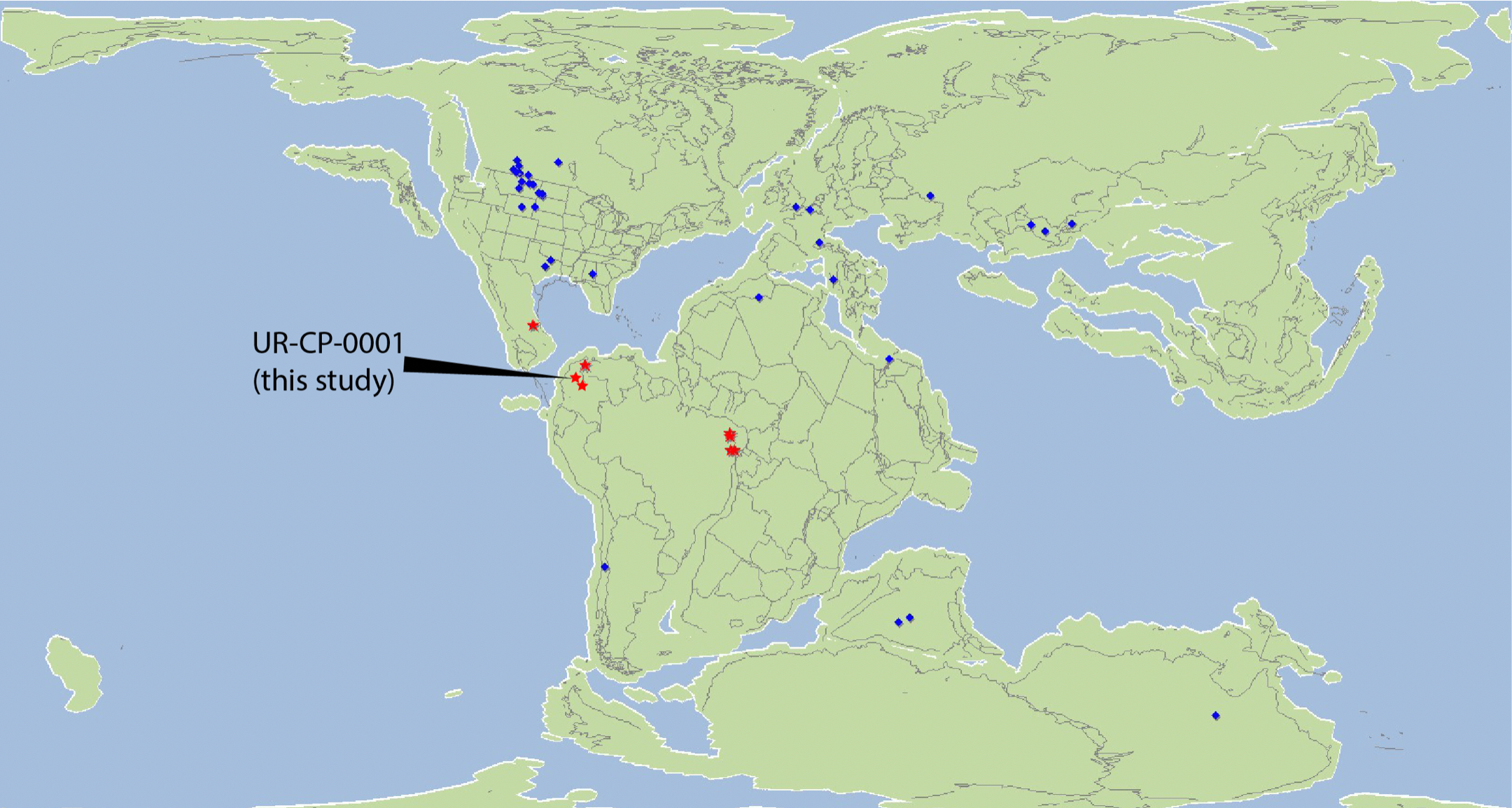

Supplement: Supplemental Information 5 — Continents reconstruction at 130 Ma, from Paleobiology Database www.fossilworks.org [file peerj-08-9479-s005.png]
